# Impact of Yoga on Spinal Mobility and Psychological Outcomes in Patients with Axial Spondyloarthritis: A Prospective Non-randomized Controlled Study

**DOI:** 10.1007/s00296-026-06202-4

**Published:** 2026-06-22

**Authors:** Vanessa Bundle, Claudia Bouzas, Alp Temiz, Birte Coppers, Ines Ebner, Paloma Palm von Alten Blaskowitz, Sebastian Rudolf, Anna-Maria Liphardt, Maria Gabriella Raimondo, Andreas Wirsching, Elizabeth Araujo, Filippo Fagni, Andreas Ramming, Georg Schett, Harriet Morf

**Affiliations:** 1 Department of Medicine 3 Rheumatology and Immunology, Friedrich-Alexander-Universität Erlangen-Nürnberg, Uniklinikum Erlangen, Erlangen, Germany; 2 Deutsches Zentrum Für Immuntherapie (DZI), Friedrich-Alexander-Universität Erlangen-Nürnberg, Uniklinikum Erlangen, Erlangen, Germany

**Keywords:** Spondyloarthritis, Axial spondyloarthritis, Yoga, Physical therapy modalities, Range of motion, Kinesiophobia, Quality of life

## Abstract

Exercise is a key non-pharmacological component of therapy in axial spondyloarthritis (axSpA). Evidence for yoga as a mind–body intervention remains limited. This study aimed to assess the effects of an 8-week yoga program on mobility, function, disease activity, activity-related competences, and psychological outcomes in axSpA compared with physiotherapy and healthy individuals. We conducted a prospective, non-randomized, study (University Hospital Erlangen, May 2021–January 2024). AxSpA patients participated either in weekly yoga sessions for 8 weeks (approximately 60 min; online format) or received standard individual physiotherapy (≥ 30 min/week). A healthy control group completed the same yoga program. Health related outcomes at baseline and follow-up were measured and changes were analysed using linear mixed-effects models. Seventy-six participants were included. In axSpA, spinal mobility improved in the yoga group (BASMI Δ − 0.31; p = 0.002), while the physiotherapy group worsened (Δ + 0.29; p = 0.003). Physical activity related health competency (control competence Δ + 1.10; p = 0.001; self-regulation Δ + 0.72; p = 0.021) and psychological outcomes improved in the yoga group with increased quality of life mental scores (Δ + 6.62; p < 0.001) and reduced kinesiophobia (TSK Δ − 3.25; p = 0.008); physiotherapy showed no meaningful changes. Disease activity showed a significant decrease in the yoga group (BASDAI Δ –0.44; p = 0.049). Healthy individuals showed similar directional changes with smaller effects. An 8-week yoga intervention was associated with improvements in mobility, psychological outcomes and activity-related competences. Findings support yoga as a complementary treatment addressing both physical and psychological dimensions of axSpA. The study was registered in the German Clinical Trials Register (DRKS-ID: DRKS00025215).

**Supplementary Information:**

The online version contains supplementary material available at 10.1007/s00296-026-06202-4.

## Introduction

Axial spondyloarthritis (axSpA) is a chronic inflammatory disease, which can cause inflammatory back pain, morning stiffness, and reduced mobility, often accompanied by fatigue and reduced physical functioning. AxSpA affects different joints, especially the spine [1]. Beyond musculoskeletal symptoms, axSpA is associated with decreased quality of life, work participation, and psychological well-being [2, 3]. Therefore, preservation of spinal mobility and physical function is a central therapeutic goal, alongside effective control of inflammatory disease activity.

Current management strategies reflect the multidimensional nature of axSpA. According to the 2022 ASAS-EULAR recommendations, optimal care includes a combination of pharmacological and non-pharmacological treatments, with regular exercise and patient education being essential components of disease management [4, 5].

While pharmacological therapy aims primarily at controlling inflammation and preventing structural damage, non-pharmacological interventions address functional limitations, secondary impairments, and long-term disability. Exercise therapy has been shown to improve disease-specific outcomes such as physical function, spinal mobility, pain, and stiffness in axSpA, while also supporting self-management and physical activity participation [6–8].

Systematic reviews and meta-analyses of randomized controlled trials demonstrate significant improvements in functional ability measured by the Bath Ankylosing Spondylitis Functional Index (BASFI), spinal mobility measured by the Bath Ankylosing Spondylitis Metrology Index (BASMI), and disease activity measured by the Bath Ankylosing Spondylitis Disease Activity Index (BASDAI) following exercise interventions [7–9].

Physiotherapy-based exercise programs represent an evidence-based standard of non-pharmacological care in axSpA. Several randomized controlled trials and systematic reviews have shown that physiotherapy can improve spinal mobility, reduce pain and stiffness, and positively influence global health perception [10, 11].

Yoga as a complementary therapy with focus on physical and psychological aspects, is a mind–body practice with Indian origins [12], that includes different parts, such as breathing techniques, exercises called Asanas and meditation [13]. A few studies have shown that yoga can reduce back pain [14, 15] and improve mobility [16, 17] in healthy people and in patients with rheumatic diseases [12].

A recent systematic review and meta-analysis of mind–body exercise interventions, including yoga, tai chi, pilates, and qigong, reported beneficial effects on physical function, disease activity, and quality of life in patients with axSpA, although heterogeneity of interventions and methodical limitations warrant cautious interpretation [18].

Evidence specifically addressing yoga in axSpA has expanded in recent years, including remote delivery models. Randomized controlled trials investigating tele-yoga interventions have demonstrated improvements in physical function, disease activity, and selected quality-of-life outcomes compared with control conditions [19, 20]. These findings are clinically relevant, as remote and home-based delivery may reduce barriers to participation and support long-term adherence to exercise recommendations. Digital exercise support has also been associated with higher home-based exercise frequency in patients with axSpA [21].

Beyond physical impairments, psychological factors play an important role in functional outcomes in axSpA. Fear of movement (kinesiophobia) has emerged as a clinically relevant construct, as it may contribute to avoidance behaviour, reduced physical activity, and impaired quality of life. Recent studies indicate that kinesiophobia is more prevalent in axSpA than in healthy populations and is associated with worse patient-reported outcomes [22]. Furthermore, kinesiophobia appears to be a part of a psychological burden in axSpA, including emotional distress and reduced health-related quality of life [23]. Interventions that promote safe movement therapy and enhance confidence in physical activity may therefore have meaningful physical and psychological effects. Also, in other chronic diseases such as Parkinson’s disease or breast cancer the positive effect of yoga on mental health as reducing fatigue, sleep disturbances, anxiety and depression is proven [24–26].

So far, previous work has summarized the potential role of yoga in rheumatic diseases more broadly [12]. Despite the growing evidence supporting exercise and yoga-based interventions in axSpA, important gaps remain. The present study adds prospective controlled data specifically in axSpA by directly comparing an eight-week yoga intervention with standard physiotherapy and by including both psychological outcomes and a healthy reference group undergoing the same yoga protocol.

## Methods

### Study design and patient recruitment

This prospective, non-randomized interventional controlled trial was designed to compare a yoga-intervention-group with a physiotherapy control group in patients with axSpA. Participants were assigned at the individual level. Group allocation was non-randomized and based on patient preference and on whether participants were already receiving physiotherapy. Reporting was guided by the STROBE reporting checklist; the completed checklist is provided as supplementary material. The patients were recruited between May 2021 and March 2023. All patients were recruited from the outpatient clinics of the Department of Rheumatology and Immunology at the University Hospital Erlangen in Germany. Male and female patients between 18 and 70 years of age with axSpA were eligible for inclusion. These patients had to be on stable immunosuppressive therapy for the entire duration of the study. The exclusion criteria were complete ankylosis of the spine and pregnancy. Healthy participants were recruited separately by study staff between May 2023 and August 2023. Inclusion criteria were an age between 18 and 70 years. Individuals with rheumatic disease or other relevant diseases that affect spinal mobility, as well as pregnant individuals, were excluded.

### Complementary treatments

The control group received individual physiotherapy sessions as standard of care with a minimum of 30 min per week, with participants either continuing previously prescribed physiotherapy or initiating treatment at study entry. Physiotherapy reflected routine standard care and was not standardized beyond the minimum frequency and duration; content was determined individually by the treating physiotherapist according to clinical needs. This reflects real world data, as there is no standardized physiotherapy protocol for axSpA in Germany. The yoga intervention consisted of weekly yoga sessions, for eight consecutive weeks and was based on a mind–body approach combining physical movement, breathing techniques, body awareness and relaxation. The program included exercises from Hatha Yoga like modified salutation, stretching poses, twist and backbends. The protocol was specifically designed to accommodate the physical limitations of rheumatic patients, prioritizing joint protection and improving axial mobility. The sessions aimed to improve mobility, strength, balance, and mental relaxation at different difficulty levels, so that all patients could perform the guided yoga techniques. Exercises were repeated according to individual ability, and progression was achieved by offering different difficulty levels and modifications rather than by increasing intensity uniformly. Yoga sessions were delivered by a trained yoga instructor experienced in adapting exercises for patients with rheumatic diseases. Each session lasted approximately 60 min and was conducted via Zoom or through pre-recorded videos, allowing participants to follow the program flexibly. Participation was verified by camera, and reminders were sent if patients were absent. Adherence was measured with a presence list; after not attending the class for two times the patients were contacted via email. Additionally, the patients were instructed to practice parts of the yoga session during the week, or to repeat the whole session using the recorded materials provided by email (either pre-recorded videos or recordings of the live Zoom session). Healthy individuals completed the same yoga protocol, also lasting approximately 60 min, once a week, for eight consecutive weeks.

### Ethical considerations

The study protocol was registered in the German Clinical Trials Register (DRKS- ID: DRKS00025215) and approved by the ethics committee of the medical faculty of the Friedrich-Alexander-Universität Erlangen-Nürnberg (ethics committee reference number: 349_20B, date of the vote: 29.09.2020) [27]. The participation in the study was voluntary, participation in the study was only possible after signing the informed consent form. The patient data were pseudonymized. The collected data were stored and analyzed in a password-protected database called REDCap (Research Electronic Data Capture) [28]. Patients had the possibility to withdraw from the study at any time, upon which all personal data would be deleted. The study was conducted in accordance with the Declaration of Helsinki and written informed consent was obtained from all participants.

### Measurements

All attendees underwent baseline measurements and answered questionnaires before the start of the study. The questionnaires were collected in paper form and were transferred to REDCap and consisted of the BASDAI, BASFI, BSA, PAHCO, SF-36, TSK, and a self-developed questionnaire focusing on physiotherapy [29, 31–34]. Main outcomes included spinal mobility assessed by BASMI and patient-reported physical, disease-related, activity-related, and psychological outcomes assessed by BASFI, BASDAI, BSA, PAHCO, SF-36, and TSK. The BSA questionnaire assesses work-, leisure- and sport-related activity; work-related activity was assessed using a categorical score, while leisure- and sport-related activity were expressed as minutes per week (min/week). The TSK was analysed both as a continuous score (TSK2) and using a dichotomized classification (cut-off > 37). Clinical parameters were collected from the patients' clinical records (CRP, BMI, age, HLA-B27 status, diagnosis).

Spinal mobility was assessed using the BASMI (Bath Ankylosing Spondylitis Metrology Index), which consists of five separate measurements of the spine [29, 30]. Eight to twelve weeks after baseline assessment, participants received follow-up measurements with all questionnaires and the BASMI assessment. An overview of the questionnaires and measurements is provided in Table 1.

**Table 1:** Questionnaires and measurements

Questionnaires	Abbreviation	Method of administration	Range	Purpose/content	References
Bath Ankylosing Spondylitis Metrology Index	BASMI	Physical measures	0–10 (higher scores indicate worse spinal mobility)	Spinal mobility	[29, 30]
Bath Ankylosing Spondylitis Disease Activity Index	BASDAI	Self-report	0–10 (higher scores indicate higher disease activity)	Measures disease activity	[29]
Bath Ankylosing Spondylitis Functional Index	BASFI	Self-report	0–10 (higher scores indicate greater functional impairment)	Measures functional status	[29]
Bewegungs- und Sportaktivität	BSA	Self-report	BSA Work: score 0–9 (higher scores indicate higher activity) BSA Leisure & Sport: expressed as minutes per week (min/week)	Measurement of physical and sports activities	[34]
Physical activity-related health competence	PAHCO	Self-report	Movement competence: 0–17.6 Control competence: 0–10.8 Self-regulation competence: 0–14.8 higher scores indicate better competence	Measures personal competencies for a healthy, physically active lifestyle	[31]
Short Form 36 Questionnaire	SF-36	Self-report	0–100: higher score indicates a better health status	Health-related quality of life, 36 items that assess physical and psychological health	[32]
The Tampa Scale of Kinesiophobia	TSK	Self-report	17–68: 17–37 = low fear of movement > 37 = high fear of movement	Measures fear of movement and (re)injury; assesses kinesiophobia related to pain and physical activity avoidance	[33]

### Statistical analysis

Data were analyzed at two time points (baseline and follow-up). Baseline characteristics were summarized descriptively. Categorical variables are reported as n (%), and continuous variables as mean (SD). Group comparisons were performed using Pearson’s chi-squared test or Fisher’s exact test for categorical variables and the Kruskal–Wallis rank-sum test for continuous variables. Longitudinal changes in outcomes were analyzed using linear mixed-effects models with a random intercept for each participant and fixed effects for time (baseline vs follow-up), group, and their interaction. As a sensitivity analysis, baseline values of the respective outcome variables were additionally included as covariates in the linear mixed-effects models to account for baseline differences between groups. Estimated marginal means with 95% confidence intervals were derived for each group and time point. Changes from baseline to follow-up were expressed as Δ values (follow-up minus baseline). Missing data were not imputed; analyses were based on available data for each outcome. Within-group and between-group contrasts were obtained from the fitted models. To explore associations between clinical variables and outcome changes, linear regression analyses were performed separately within each group. Covariates included CRP (baseline and Δ), BMI, age, HLA-B27 status, and diagnosis. Subgroup comparisons were conducted using parametric ANOVA with Bonferroni-adjusted post hoc tests. All tests were two-sided, and p-values < 0.05 were considered statistically significant. All statistical analyses were performed using R version 4.5.1.

## Results

### Patient characteristics

Overall, seventy-six participants were included in the study and assigned to one of three groups (see Flow-Chart in the supplementary material): axSpA yoga (n = 24), axSpA physiotherapy (n = 24), and healthy yoga (n = 28). Follow-up assessments were conducted after 8–12 weeks. Analyses were performed based on available data for each outcome.

The baseline characteristics are summarized in Table 2.

**Table 2:** Patient characteristics (baseline)

Patient characteristics	Control axSpA Physiotherapy (n = 24)	Intervention axSpA yoga (n = 24)	Intervention healthy yoga (n = 28)	p-value (between all groups)
Age [years] mean ± SD	47 ± 11	44 ± 14	43 ± 15	p = 0.60
Sex female number (%)	17 (71%)	19 (79%)	26 (93%)	p = 0.13
BMI [kg/m2] mean ± SD	27.2 ± 5.0	23.9 ± 3.2	26.0 ± 7.3	p = 0.20
BASMI mean ± SD	1.5 ± 1.4	1.9 ± 1.2	2.8 ± 0.6	p < 0.001
BASDAI mean ± SD	4.7 ± 2.1	3.9 ± 2.7	1.4 ± 1.4	p < 0.001
BASFI mean ± SD	3.6 ± 2.2	2.7 ± 2.4	0.3 ± 0.3	p < 0.001

Patient characteristics of the intervention group with axSpA and the intervention healthy group (both using yoga) and the control group (using physiotherapy) in a prospective, non-randomized controlled trial with 76 (81.6% female, 18.4% male) in Erlangen, Germany, from May 2021 to January 2024

*BMI *Body mass index*, BASMI *Bath ankylosing spondylitis metrology index*, BASDAI *Bath ankylosing spondylitis disease activity index,* BASFI *Bath ankylosing spondylitis functional index

## Mobility and physical function

### Comparison of yoga-treated patients and physiotherapy

Baseline characteristics did not differ significantly between the intervention and control groups in age, sex, or BMI. Furthermore, the patient yoga group and physiotherapy groups did not differ significantly at baseline in spinal mobility (BASMI p = 0.643), physical function (BASFI p = 0.844), BSA or the SF-36 physical score (p = 0.887) After the eight-week yoga intervention, the BASMI (Bath Ankylosing Spondylitis Metrology Index) decreased significantly (∆ = −0.31; p = 0.002), indicating an improvement in mobility, as lower BASMI scores reflect better functional range. The physiotherapy group showed a significant increase in BASMI (∆ = + 0.29; p = 0.003), consistent with reduced spinal mobility.

The Bath Ankylosing Spondylitis Functional Index (BASFI) showed only small, nonsignificant changes in both groups. BASFI slightly decreased in the yoga group (∆ = −0.17; p = 0.424), indicating a minor reduction in functional limitations and slightly increased in the physiotherapy group (∆ = + 0.05; p = 0.818).

Physical activity levels, measured by the BSA questionnaire, did not change significantly in either group. Work-related activity remained largely stable over time and did not reach statistical significance (yoga: ∆ = + 0.09; p = 0.799; physiotherapy: ∆ = −0.5; p = 0.132). Leisure-activity showed a small, statistically non-significant decrease in the intervention group (∆ = −404.46 min/week; p = 0.054) and remained stable in the control group (∆ = −33.34 min/week; p = 0.871). Sport-related activity showed a non-significant increase in the yoga group (∆ = + 57.29 min/week; p = 0.061) and remained largely stable in the control group (∆ = −12.49 min/week; p = 0.694).

**Fig. 1:**
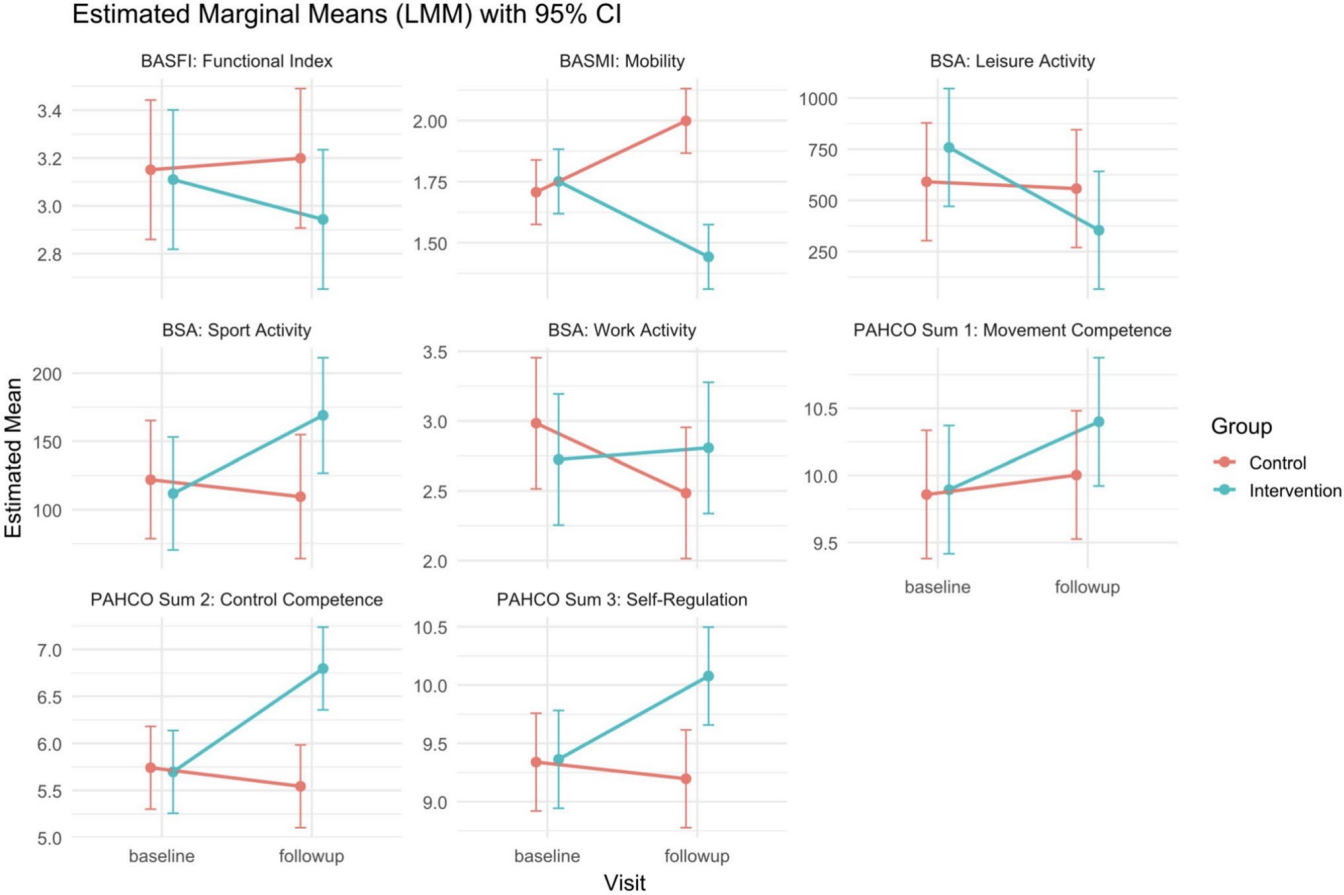
Estimated marginal means (EMMs) for mobility and activity outcomes (Yoga patients vs. Physiotherapy patients)

Baseline PAHCO values did not differ significantly between the intervention and control group at baseline (movement competence: p = 0.917; control competence: p = 0.889; self-regulation: p = 0.937). In the yoga group, control competence and self-regulation improved significantly (∆control competence = + 1.10; p = 0.001; ∆self-regulation = + 0.72; p = 0.021), while the movement competence showed a small, non-significant increase (∆ = + 0.51; p = 0.144). In the physiotherapy group, no significant changes were observed across PAHCO subscales. All results are shown in Fig. 1.

The physical component of the SF-36 remained largely stable in both groups, despite a small, non-significant increase in the physiotherapy group and a small, non-significant decrease in the yoga group. This result is shown in Fig. 3.

### Comparison of yoga-treated patients and healthy individuals

When comparing yoga-treated patients with healthy individuals who completed the same yoga protocol, differences in mobility and functional ability were observed at baseline.

Baseline BASMI values were significantly higher in healthy individuals compared to yoga-treated patients (p = 0.001). Baseline BASFI values were lower in the group of healthy individuals, indicating better physical function. Baseline PAHCO scores were generally higher in the healthy cohort, suggesting greater perceived competence in dealing with physical activity. The SF-36 physical score was significantly lower in the patient group compared with healthy participants (p < 0.001), indicating poorer self-perceived physical health status.

Across the eight-week period, both groups showed largely comparable changes. For spinal mobility, BASMI decreased from baseline to follow-up in both groups. The within-group change was statistically significant in yoga-treated patients (Δ = − 0.31; p = 0.020), whereas the change in healthy individuals was not statistically significant (∆ = –0.21; p = 0.101). Physical function, assessed by BASFI, remained stable in both cohorts. Changes from baseline to follow-up were small and not statistically significant, but showed a small decrease in both groups, indicating a slight increase in physical function.

Regarding activity-related health competences (PAHCO), yoga-treated patients showed a significant increase in control competence (∆ = + 1.10; p = 0.014), whereas changes in self-regulation varied depending on the comparison group (∆ = + 0.72; p = 0.078). Notably, within-group p-values for yoga-treated patients differ slightly between models due to different comparison groups and variance estimates. Movement competence demonstrated a small, non-significant improvement in the patients' group (∆ = + 0.51; p = 0.263).

In healthy individuals, control competence also improved significantly (∆ = + 0.89; p = 0.037), whereas self-regulation remained largely unchanged (∆ = −0.02; p = 0.959). Movement competence demonstrated a small, non-significant improvement, similar to the patient group (∆ = + 0.49; p = 0.257).

**Fig. 2:**
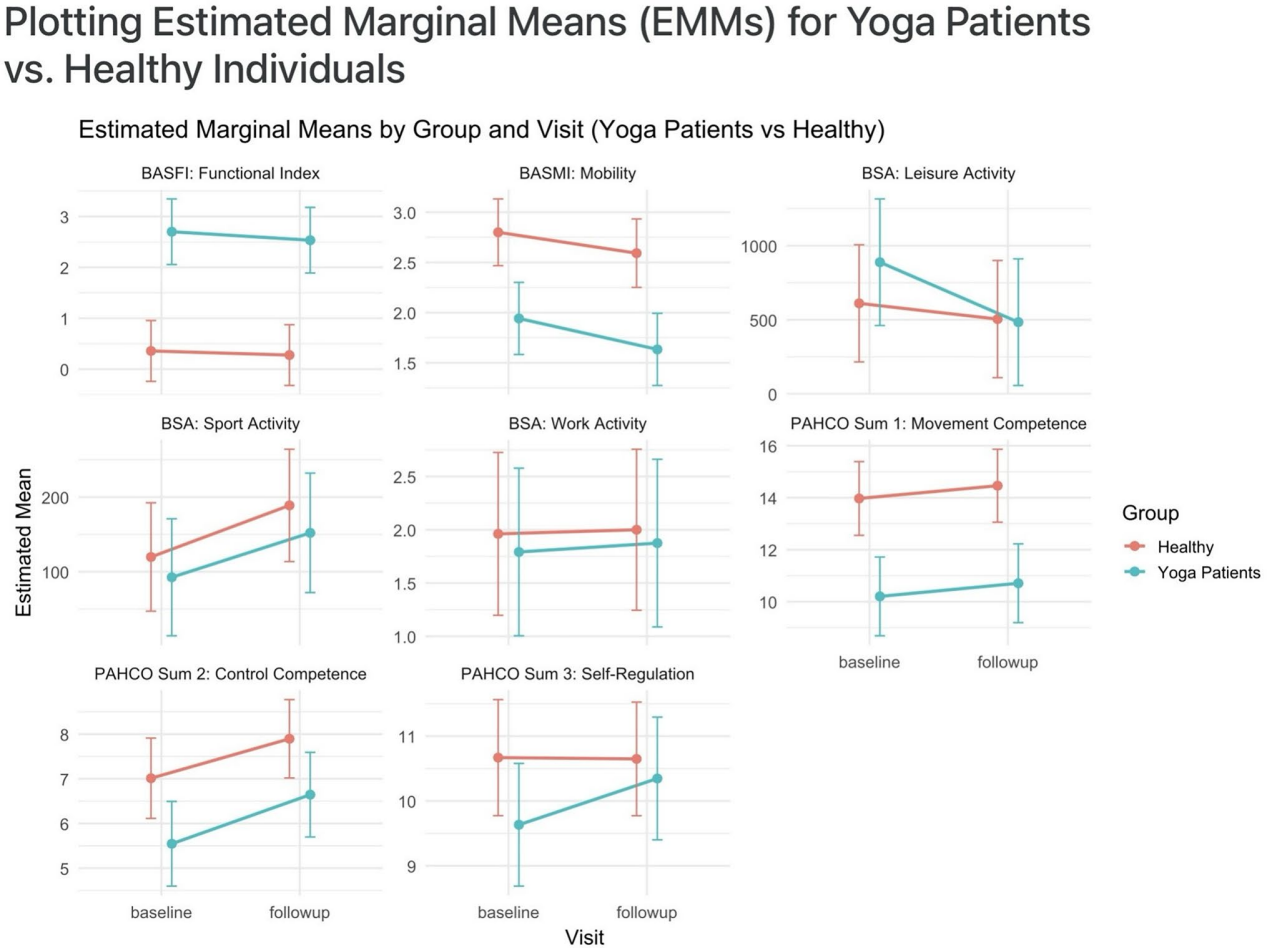
Estimated marginal means (EMMs) for mobility and activity outcomes (Yoga patients vs. Healthy individuals)

Physical activity levels assessed by the BSA did not reach a statistically significant change in both groups. Sport-related activity increased in both groups, but variability was high and confidence intervals overlapped. Leisure Activity decreased in both groups, while work activity remained largely stable. All results are shown in Fig. 2.

The SF-36 physical component remained largely stable in both yoga-treated groups, indicating no short-term change in self-perceived physical health-related quality of life. This result is shown in Fig. 4.

## Psychological outcomes and disease activity

### Comparison of yoga-treated patients and physiotherapy

At baseline, no statistically significant differences were observed between the yoga and physiotherapy groups, indicating comparable psychological and disease-related status prior to the intervention.

Disease activity, assessed by the Bath Ankylosing Spondylitis Disease Activity Index (BASDAI), decreased significant ly in the yoga group from baseline to follow-up (∆ = −0.44; p = 0.049), indicating a reduction in perceived disease activity. The patients receiving physiotherapy showed a smaller decrease, statistically non-significant (∆ = −0.22; p = 0.328).

Health-related quality of life, measured by the psychological component of the SF-36, improved significantly in the yoga group (∆ = + 6.62; p < 0.001), consistent with improved psychological well-being. No significant change in the psychological SF-36 domain was observed in the physiotherapy group (∆ = + 0.63; p = 0.676).

Kinesiophobia decreased in the continuous TSK significantly in the yoga group (TSK2 ∆ = −3.25; p = 0.008), consistent with a reduction in fear of movement. In contrast, TSK2-scores in the physiotherapy group showed a small, non-significant increase from baseline to follow-up (∆ = + 0.79; p = 0.503).

**Fig. 3:**
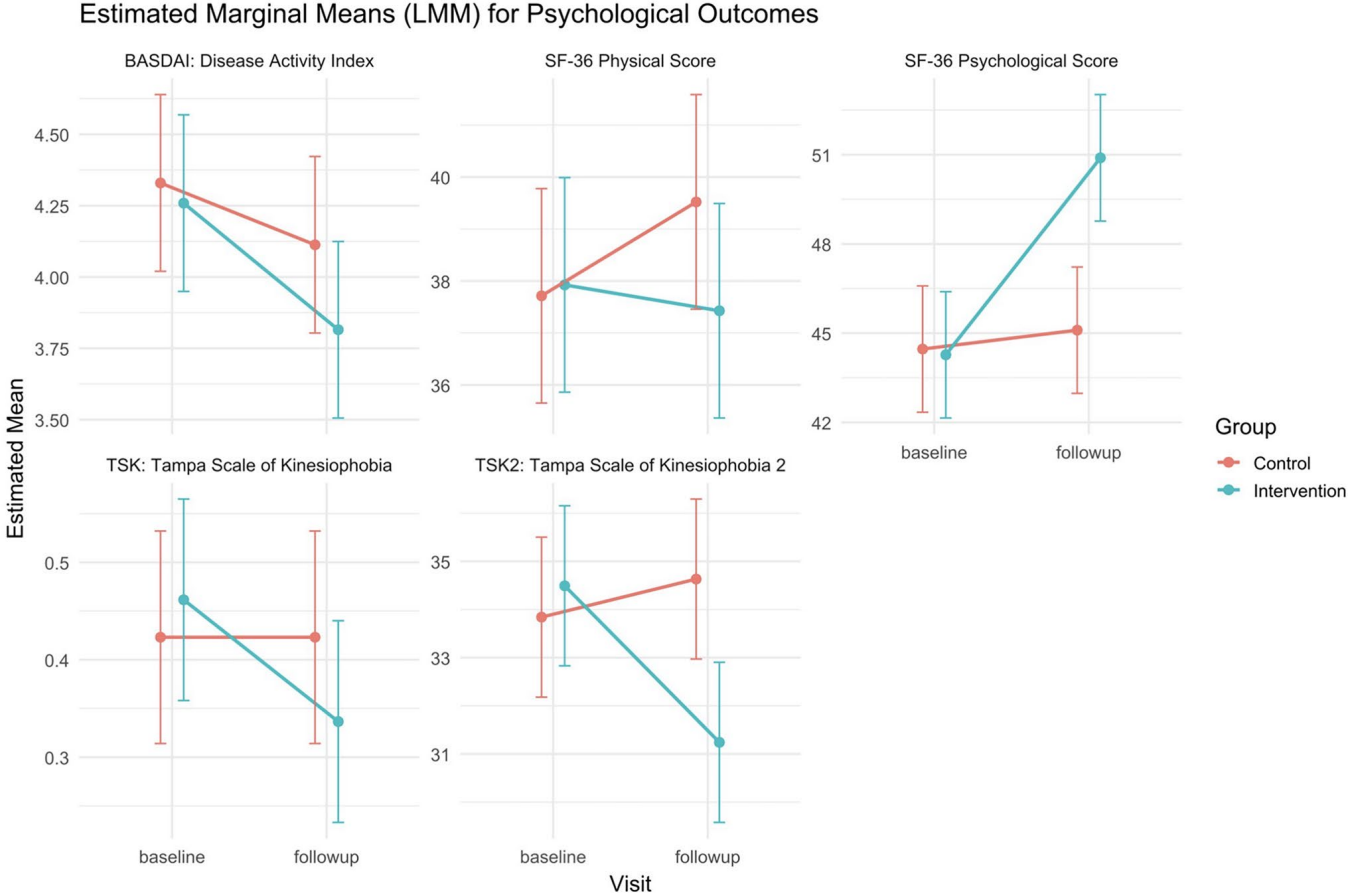
Estimated marginal means (EMMs) for psychological outcomes, disease activity and the physical SF-36 score (Yoga patients vs. physiotherapy patients)

Overall, favorable changes in psychological wellbeing and movement-related fear were observed predominantly in the yoga group, whereas physiotherapy was not associated with significant changes in these psychological outcomes. All results are shown in Fig. 3.

### Comparison of yoga-treated patients and healthy individuals

At baseline, yoga-treated patients differed significantly from healthy individuals in several categories. Patients reported higher disease burden (BASDAI; p < 0.001) and higher Kinesiophobia as reflected by higher TSK-scores (p < 0.001).

The baseline psychological well-being (SF-36 Psychological score) did not differ significantly between groups (p = 0.417).

Over the intervention period, yoga-treated patients demonstrated a significant improvement in psychological well-being, reflected by an increase in the SF-36 psychological score from baseline to follow-up (∆ = + 6.62; p < 0.001). In healthy individuals, only a small, non-significant increase was observed (∆ = + 1.02; p = 0.566), indicating stable psychological well-being.

Kinesiophobia decreased significantly in yoga-treated patients (TSK2 ∆ = −3.25; p = 0.001), indicating reduced fear of movement. In contrast, healthy individuals demonstrated a significant increase in TSK2 scores over the same period (∆ = + 3.47; p < 0.001), meanwhile this increase was not reflected in the dichotomized TSK classification (cut-off > 37).

**Fig. 4:**
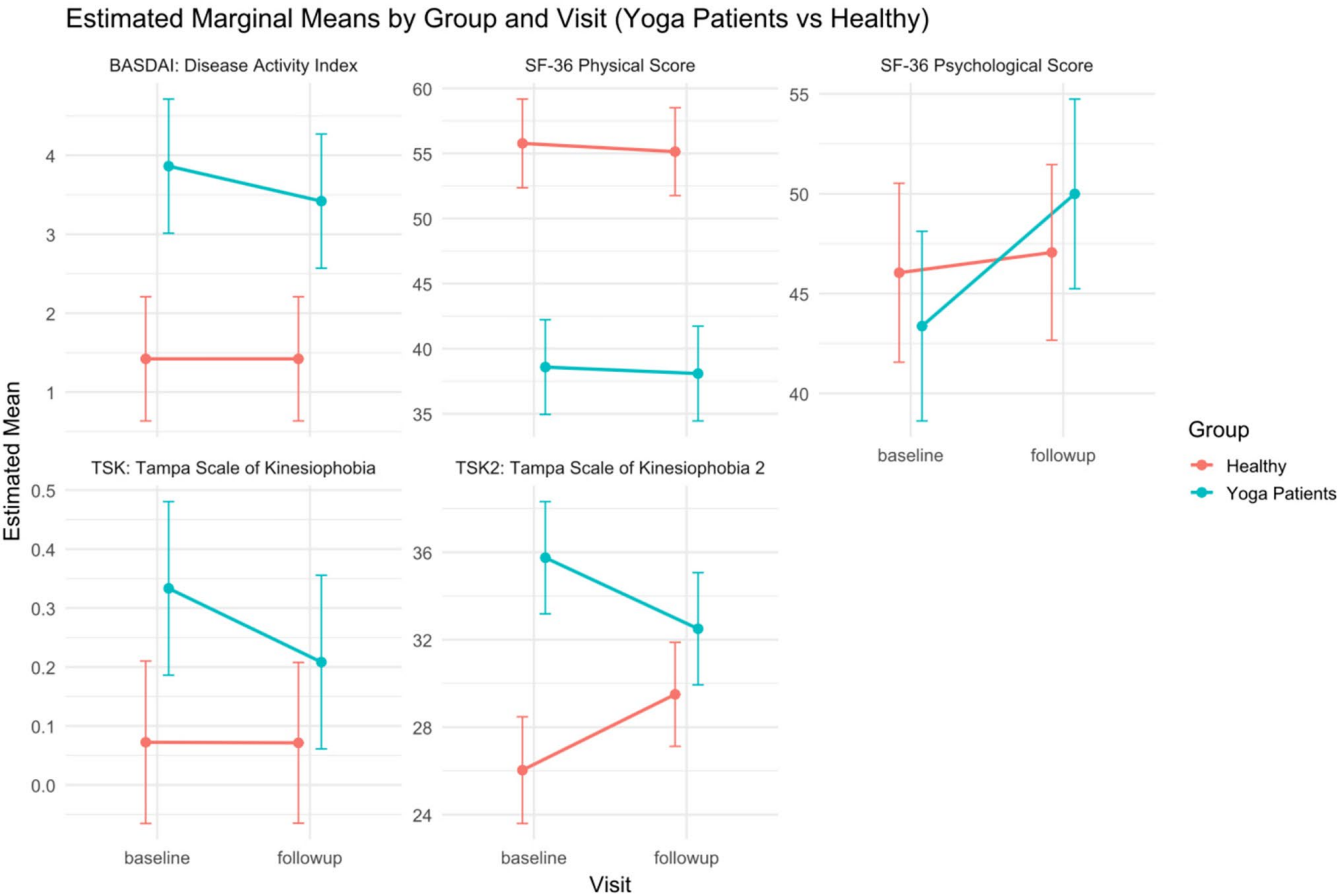
Estimated marginal means (EMMs) for psychological outcomes, disease activity and the physical SF-36 score (Yoga patients vs. Healthy individuals)

Disease activity assessed by BASDAI decreased in yoga-treated patients (∆ = −0.44), although this change did not reach statistical significance (p = 0.107). As expected, BASDAI scores in healthy participants remained unchanged over time. All results are shown in Fig. 4.

### Adverse events

No intervention-related adverse events were reported to the study team.

## Discussion

This prospective, non-randomized controlled study evaluated the effects of an eight-week yoga intervention on mobility, physical function, activity behavior, and psychological outcomes in patients with axSpA, compared with a physiotherapy control group and with healthy individuals participating the same yoga protocol. The non-randomized design may have introduced selection bias, as participants were not randomly allocated to intervention groups.

The central finding is that yoga was associated with improved spinal mobility (BASMI), while the physiotherapy group showed a small decrease.

In contrast, self-reported physical function (BASFI) and the physical component of health-related quality of life (SF-36 physical) remained largely unchanged. The most pronounced effects beyond mobility were observed in the psychological domain: psychological well-being (SF-36 psychological) improved significantly in the yoga patient group, and kinesiophobia (TSK/TSK2) decreased significantly, whereas physiotherapy did not show comparable changes. In addition, PAHCO improved primarily in the yoga group, suggesting enhanced self-regulation and control of physical activity behavior.

Taken together, these findings suggest that a structured yoga intervention may be associated with clinically meaningful changes in axSpA, particularly for mobility and selected psychological determinants. Psychological factors such as kinesiophobia and movement-related beliefs are increasingly recognized as important determinants of functional limitation, physical activity behavior and long-term participation in patients with axSpA [22].

Yoga practice may benefit patients not only through physical movement but also through mechanisms related to stress regulation, emotional regulation, mindfulness and self-awareness, which have been associated with improved psychological well-being [35]. Regarding mobility and physical function, preserving spinal mobility is a central treatment goal in axSpA, and regular exercise is recommended as an important non-pharmacological component of care [4]. In this study, yoga-treated patients showed a significant improvement in spinal mobility, reflected by reduced BASMI values, indicating improved functional range. While the observed change in BASMI was statistically significant, its magnitude was relatively small. However, even modest changes in spinal mobility may be clinically relevant in axSpA, particularly over a short intervention period and in contrast to the deterioration observed in the physiotherapy group, which was also of relatively small magnitude.

The direction of this improvement aligns with previous studies. A randomized controlled trial demonstrated that an eight-week tele-yoga program led to significant improvements in spinal mobility, physical function and disease activity [19], and a complementary meta-analysis of mind–body exercise supports beneficial effects on spinal mobility and physical function in axSpA [18]. Similarly, a comparative intervention study reported improvements in mobility, functional capacity, pain and sleep quality following yoga-based exercise [36]. In addition, a recent systematic review and meta-analysis of randomized controlled trials on exercise prescription in axSpA confirmed beneficial effects of exercise interventions on disease activity, physical function, spinal mobility, thoracic expansion, and fatigue, supporting the clinical relevance of structured exercise as a core non-pharmacological treatment strategy [37].

In contrast, BASFI remained largely stable in both groups. This divergence between improved objective mobility and stable self-reported function may be explained by the multidimensional nature of BASFI, which is influenced by pain, fatigue and behavioral adaption and may require longer intervention periods to change. The yoga group showed a small decrease in BASFI, indicating reduced functional limitations, whereas the physiotherapy group showed a small increase. The lack of significant BASFI improvement, despite positive findings in other studies [18, 19, 36], can likely be explained by methodological differences. In randomized controlled trials with larger sample sizes and higher training frequency, yoga was often performed three times per week and BASFI was a primary endpoint, leading to significant improvements [19, 36]. In contrast, the present study applied a lower training dose, a non-randomized design and a moderate sample size, which may have limited statistical power to detect smaller changes in patient-reported outcomes.

Initially, the physiotherapy findings may appear counterintuitive, because physiotherapy is widely recommended in axSpA care. However, physiotherapy is not a uniform intervention, and its effectiveness depends on the type and intensity of the program. A systematic review and meta-analysis indicate that outcomes vary according to intervention type and intensity, with supervised, exercise-based physiotherapy showing clearer benefits than usual care or heterogeneous approaches [7] In routine clinical practice, physiotherapy may include passive components such as massage or therapist-guided mobilization, which limits comparability with structured exercise-based interventions such as yoga. Active, regularly performed mobility exercises appear to be essential for improving spinal mobility, which may have contributed the lack of improvement observed in the physiotherapy group. The observed increase in BASMI scores in the physiotherapy group should therefore be interpreted cautiously and may be related to intervention heterogeneity, differences in active mobility-focused exercise, adherence-related factors, or potential assessor variability.

Regarding physical activity behavior and activity-related competences, overall physical activity levels assessed by the BSA questionnaire did not change significantly. However, relevant patterns were observed in the yoga group, where sport-related activity increased and leisure activity decreased. This suggests a reorganization of activity behavior rather than an overall increase in activity volume. This interpretation is consistent with the concept of physical activity substitution, where structured exercise replaces other forms of activity [38, 39]

PAHCO showed significant improvements in control competence and self-regulation in the yoga group. These competences are conceptualized as key prerequisites for sustainable physical activity behavior^31^]. Empirical PAHCO-based research has further demonstrated that these competences are associated with higher levels of physical activity [40]. In patients with axSpA, physical activity itself has been linked to improved functional and clinical outcomes, highlighting the relevance of behavioral determinants for disease management [41]. Recent data further support the relevance of motivational and self-management-related factors, showing that exercise adherence in ankylosing spondylitis is associated with perceived exercise benefits and spinal mobility [42]. Taken together, these findings suggest that changes in control competence and self-regulation may represent relevant qualitative adaptations, even in the absence of short-term increases in overall activity volume.

Regarding psychological outcomes, kinesiophobia and disease activity, another important outcome of this study was observed in the psychological domain. The SF-36 psychological component improved significantly in the yoga group, accompanied by a significant reduction in kinesiophobia. This is clinically relevant, as kinesiophobia has been shown to correlate strongly with physical function and overall health in axSpA [22], and is associated with reduced quality of life [23]. This supports the relevance of considering both psychological and physical dimensions.

In contrast, the SF-36 physical component showed no significant change. This does not contradict the observed improvements in disease-specific physical parameters but reflects known measurement characteristics of the SF-36. The physical component summary represents a broad, generic construct of perceived physical health, integrating pain, physical functioning and role limitations and is less sensitive to short-term and domain-specific changes. Accordingly, short-term improvements in specific physical domains may not be reflected in global physical health perception as captured by the SF-36 physical component [43].

The observed psychological improvements are consistent with evidence that mind–body interventions may positively influence patient-reported outcomes in addition to physical exercise components[18]. Randomized controlled trials of (tele-) yoga in axSpA have also demonstrated improvements in quality of life and symptom-related outcomes, supporting effects beyond physical function [20].

Mechanistically, yoga may address kinesiophobia through additional mind–body components, including stress regulation, body awareness, and cognitive appraisal of bodily sensations.

Direct comparative evidence between yoga and conventional exercise-based interventions in axSpA is limited. A recent controlled study comparing yoga with a structured exercise program in axSpA patients demonstrated comparable improvements in physical outcomes such as mobility, function and pain [36], suggesting that yoga can achieve similar physical benefits to conventional exercise-based therapy. Beyond these physical effects, the present study suggests potential additional effects of yoga in psychological domains. This indicates that while physical adaptations may be comparable across different exercise modalities, yoga's integrative mind–body components may be particularly relevant for addressing movement-related fear and psychological well-being. Further controlled studies focusing on psychological outcomes are needed to confirm these findings.

Disease activity (BASDAI) showed a small but statistically significant decrease in the yoga group in the baseline-adjusted analysis. However, the magnitude of change was modest and should be interpreted cautiously given the non-randomized design and baseline differences between groups. This may be related to the relatively short intervention period and lower training dose, as longer and more intensive exercise interventions have demonstrated clearer effects [44].

Finally, regarding the comparison with healthy individuals, baseline BASMI values were significantly higher in healthy individuals than in yoga-treated patients, which was clinically unexpected. This finding may reflect differences in recruitment and assessment procedures between cohorts and should therefore be interpreted cautiously. Furthermore, we cannot rule out the influence of routine physiotherapy or specialized spinal exercises among axSpA patients. This preexisting engagement in targeted physical activity could potentially confound the differences observed between the patient cohort and healthy controls. However, BASMI changes over time were smaller and not statistically significant in healthy individuals, whereas yoga-treated patients showed a significant improvement in spinal mobility. Despite this limitation, the inclusion of a healthy comparison group represents an additional strength of the study. Healthy participants showed similar directional trends but smaller and mostly non-significant changes, likely reflecting lower baseline impairment and limited potential for measurable improvement. This pattern may suggest that individuals without disease-related impairments show attenuated effects due to higher baseline function, limiting the potential for detectable improvements even when an intervention is effective.

The observed significant decrease in kinesiophobia in yoga-treated patients, contrasted with a significant increase in TSK2 in healthy individuals that was not reflected in the dichotomized TSK classification, may reflect heightened movement awareness or caution rather than clinically relevant kinesiophobia. Overall, the largely parallel trends in both groups provide preliminary support for the feasibility of yoga across populations, while more pronounced changes in patients suggest that intervention effects are more evident in the presence of baseline limitations. Including a healthy reference group therefore provides important context for interpreting disease-specific responsiveness.

## Limitations and future directions

The study has several limitations that should be considered when interpreting the findings. First, the non-randomized study design limits causal inference and does not fully exclude selection bias. Although baseline characteristics were largely comparable between the intervention groups, unmeasured confounding factors may have influenced the observed effects. Blinding of participants and intervention providers was not feasible due to the nature of the interventions, and blinded outcome assessment was not implemented. Second, the intervention period of eight weeks was relatively short. While this duration was sufficient to detect changes in mobility and psychological outcomes, longer intervention periods may be necessary to achieve more pronounced or sustained effects on disease activity and generic physical quality-of-life measures.

No formal sample size calculation was performed. In addition, physiotherapy reflected routine standard care and was less standardized than the yoga intervention with regard to content, intensity, and supervision, which limits direct comparability between interventions. The sample size was moderate, particularly for subgroup analyses and comparisons with healthy participants. Several outcomes were associated with relatively wide confidence intervals that partly overlapped between groups, indicating limited precision of the estimated effects and a degree of uncertainty in between-group comparisons. Incomplete data for some outcome measures may have reduced statistical power and contributed to uncertainty in individual estimates. Baseline differences in BASDAI between groups indicate that the control group may have been more severely affected at study entry, which further limits comparability and may have influenced the observed effects. To address baseline differences, additional baseline-adjusted sensitivity analyses were performed; the overall interpretation remained broadly comparable, although significance levels changed for some secondary outcomes.

Several outcomes, including physical activity and health competences, were assessed using self-reported questionnaires, which may be subject to recall and reporting bias. These factors may limit the generalizability of the findings to broader axSpA populations and to other healthcare settings.

Future studies should employ randomized controlled designs with larger sample sizes and extended follow-up periods to confirm and expand the present findings. Direct comparisons between yoga and standardized physiotherapy or other exercise-based interventions are needed, because conventional physiotherapy and yoga differ in their underlying therapeutic mechanisms, particularly with respect to psychological outcomes, which have so far received limited systematic attention in exercise-based intervention research in axSpA. The integration of objective activity measures and inflammatory markers may further help to identify patient subgroups that benefit most from mind–body interventions.

## Conclusion

The intervention was associated with improvements in spinal mobility and clinically relevant psychological outcomes, particularly reductions in kinesiophobia and improvements in mental health-related quality of life. These findings support the relevance of addressing psychological factors alongside physical function in the non-pharmacological intervention of inflammatory rheumatic diseases.

Although changes in disease activity were modest and generic physical quality-of-life measures remained largely stable, the overall pattern suggests that yoga may represent a valuable adjunct to established exercise-based approaches. The inclusion of a healthy comparison group further indicates that intervention effects may be more pronounced in individuals with baseline impairments. Taken together, the findings support further evaluation of structured yoga programs as part of multimodal care for axSpA. Larger controlled studies with longer follow-up periods are necessary to confirm these results.

## Supplementary Information

Below is the link to the electronic supplementary material.

Supplementary file1 (DOCX 147 KB)

Supplementary file2 (DOCX 45 KB)

## Data Availability

The datasets generated and/or analyzed during the current study are available from the corresponding author on reasonable request.

## Abbreviations

ASAS: Assessment of spondyloarthritis international society
axSpA: Axial spondyloarthritis
BASDAI: Bath ankylosing spondylitis disease activity index
BASFI: Bath Ankylosing spondylitis functional index
BASMI: Bath ankylosing spondylitis metrology index
BSA: Bewegungs- und sportaktivität
BMI: Body mass index
CRP: C-reactive protein
e.g.: Exempli gratia
et al.: et alia
EULAR: European alliance of associations for rheumatology
N: Number
OR: Odds ratio
PAHCO: Physical activity-related health competence
RCT: Randomized controlled trial
REDCap: Research electronic data capture
SD: Standard deviation
SF-36: Short form 36
TSK: Tampa scale of kinesiophobia

## Declarations

### Disclaimer

The authors confirm that no part of this manuscript has been copied from other sources or published elsewhere. No part of the scientific content, data analysis, interpretation of results, or conclusions was generated by artificial intelligence. AI-based assistance was used only for language editing as described below. All authors reviewed and approved the final manuscript.

### **Statement on AI-assist language editing**

Parts of this manuscript were linguistically revised and rephrased with the assistance of ChatGPT (OpenAI) for language editing, clarity, grammar, and readability. The tool was not used to generate scientific content, perform data analysis, interpret results, or draw conclusions. All AI-assisted edits were reviewed, verified, and approved by the authors. The authors take full responsibility for the content, integrity, and accuracy of the final manuscript.
