# Supplementary material for: Impact of Yoga on Spinal Mobility and Psychological Outcomes in Patients with Axial Spondyloarthritis: A Prospective Non-randomized Controlled Study
Source: Rheumatol Int. 2026 Jun 22;46(7):162. doi: 10.1007/s00296-026-06202-4 (PMC13287121; doi:10.1007/s00296-026-06202-4)
Supplement: Supplementary file 2 — Supplementary file2 (DOCX 45 KB) [file 296_2026_6202_MOESM2_ESM.docx]

**Participant Flow Diagram**

Assigned to study groups (n=96)

## Follow-Up 1

## Analysis

Lost after baseline (n=10)

Follow-up not completed or participant could not be reached

Lost after baseline (n=1)

Follow-up not completed or participant could not be reached

Lost after baseline (n=3)

Follow-up not completed or participant could not be reached

Allocated to healthy yoga group (n=35)

♦ Received baseline assessment (n=29)

♦ Did not receive baseline assessment (declined to participate) (n=6)

Allocated to axSpA yoga group (n=34)

♦ Received baseline assessment (n=34)

♦ Did not receive baseline assessment (n=0)

## Allocation

Allocated to axSpA Physiotherapy (n=27)

♦ Received baseline assessment (n=27)

♦ Did not receive baseline assessment (n=0)

Included in analysis (n=28)
♦ Excluded from analysis (n=0)

Included in analysis (n=24)
♦ Excluded from analysis (n=0)

Included in analysis (n=24)
♦ Excluded from analysis (n=0)

## Enrollment
